# Supplementary material for: Metagenomic Analysis of Liquor Starter Culture Revealed Beneficial Microbes’ Presence
Source: Foods. 2022 Dec 21;12(1):25. doi: 10.3390/foods12010025 (PMC9818921; doi:10.3390/foods12010025)

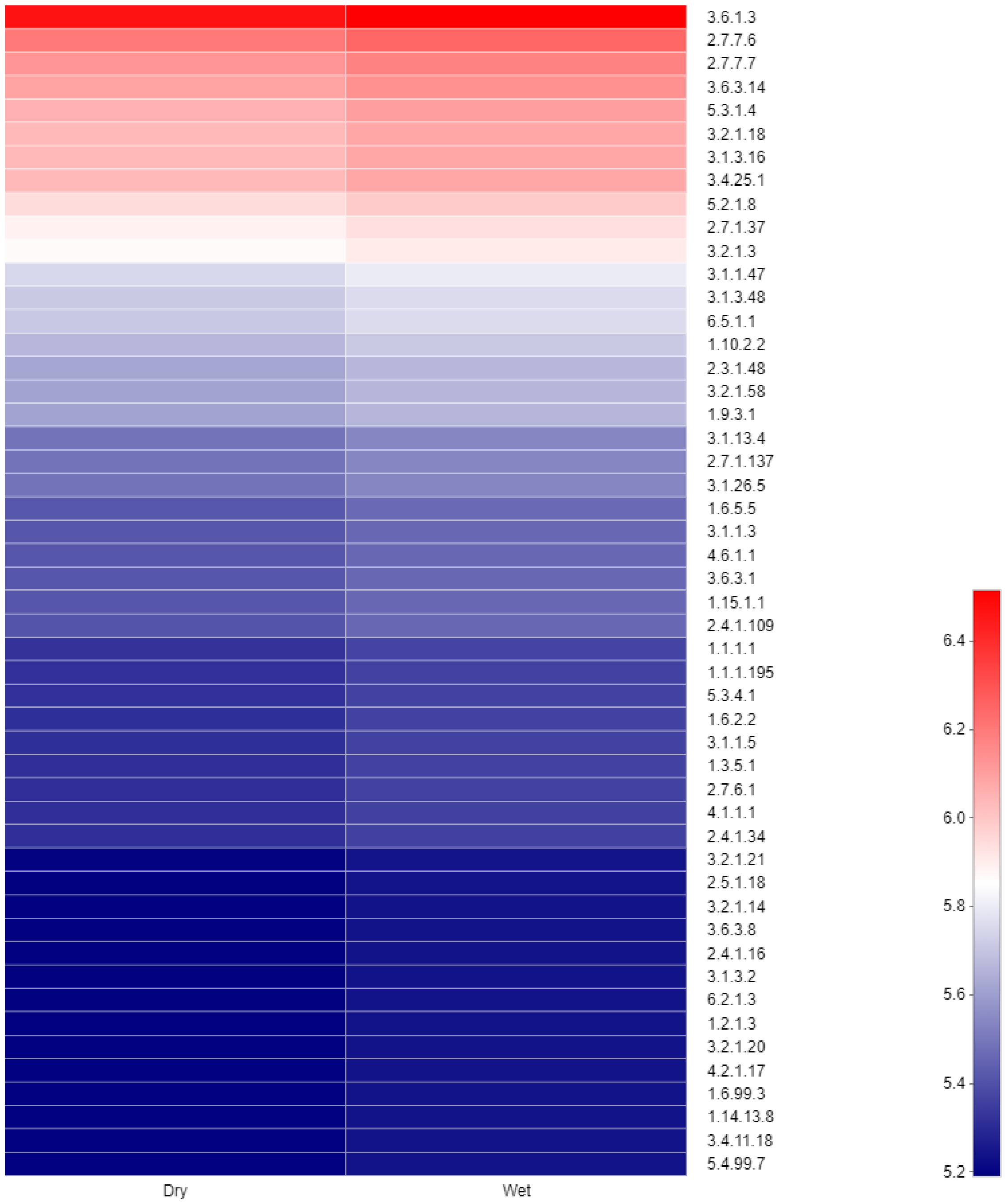

Figure S1. KEGG based Enzyme/Ko/Module Number Function Pathway Level 1. The abscissa is the sample name (or group name), and the ordinate is the Enzyme/KO/Module number or the function name of the pathway level1/2/3. The color gradient of the color block is used to display the changes in the abundance of different functions in the sample/group. The legend is the value represented by the color gradient.

Table S1. Corresponding table for the Heatmap for Figure S1.

Enzyme	Description	Dry	Wet
3.6.1.3	Adenosinetriphosphatase	2927506	3267744
2.7.7.6	DNA-directed RNA polymerase	1595717	1779578
2.7.7.7	DNA-directed DNA polymerase	1337380	1492328
3.6.3.14	H(+)-transporting two-sector ATPase	1234432	1376914
5.3.1.4	L-arabinose isomerase	1139287	1267912
3.2.1.18	Exo-alpha-sialidase	1084173	1206942
3.1.3.16	Protein-serine/threonine phosphatase	1079819	1205030
3.4.25.1	Proteasome endopeptidase complex	1079517	1204811
5.2.1.8	Peptidylprolyl isomerase	877708.7	978589.6
2.7.1.37	2.7.11.30 and 2.7.12.1	774309.7	862545.8
3.2.1.3	Glucan 1,4-alpha-glucosidase	726954.8	808548.6
3.1.1.47	1-alkyl-2-acetylglycerophosphocholine esterase	565190.1	631232.4
3.1.3.48	Protein-tyrosine-phosphatase	514839.4	574213.6
6.5.1.1	DNA ligase (ATP)	513073.6	573163.8
1.10.2.2	Quinol--cytochrome-c reductase	461514.4	515378.4
2.3.1.48	Histone acetyltransferase	416243.8	462575
3.2.1.58	Glucan 1,3-beta-glucosidase	411449.8	458842
1.9.3.1	Cytochrome-c oxidase	410050.5	458030.2
3.1.13.4	Poly(A)-specific ribonuclease	308440.3	344163
2.7.1.137	Phosphatidylinositol 3-kinase	308171.5	343953.8
3.1.26.5	Ribonuclease P	308089.2	343991.6
1.6.5.5	NADPH:quinone reductase	261329.8	290037.4

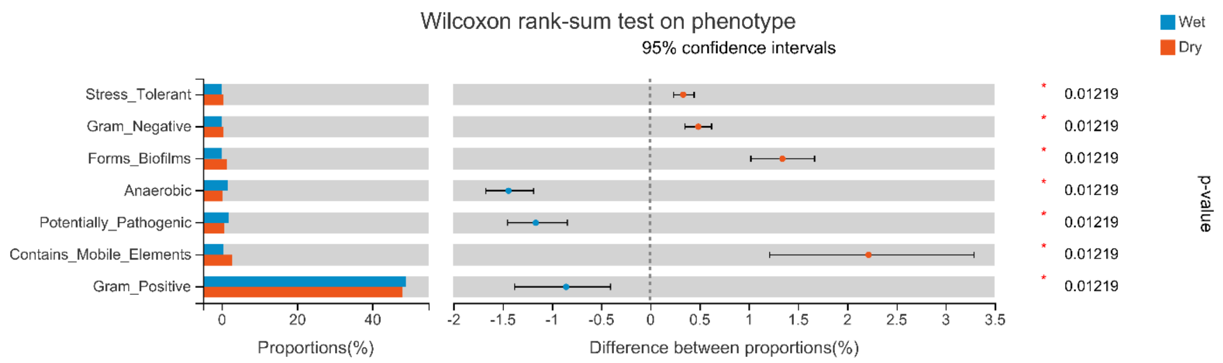

Figure S2. BugBase phenotype prediction. BugBase is capable of phenotype prediction, which phenotype types include Gram Positive, Gram Negative, Biofilm Forming, Pathogenic, Mobile Element Containing), oxygen demand (Oxygen Utilizing) and oxidative stress tolerance (Oxidative Stress Tolerant) seven categories. The horizontal axis represents the name of the phenotype, the vertical axis represents the percentage value of the relative abundance of a phenotype in the sample, and different colors represent different groups. On the far right are P values, $*0.01 < P \le 0.05$.

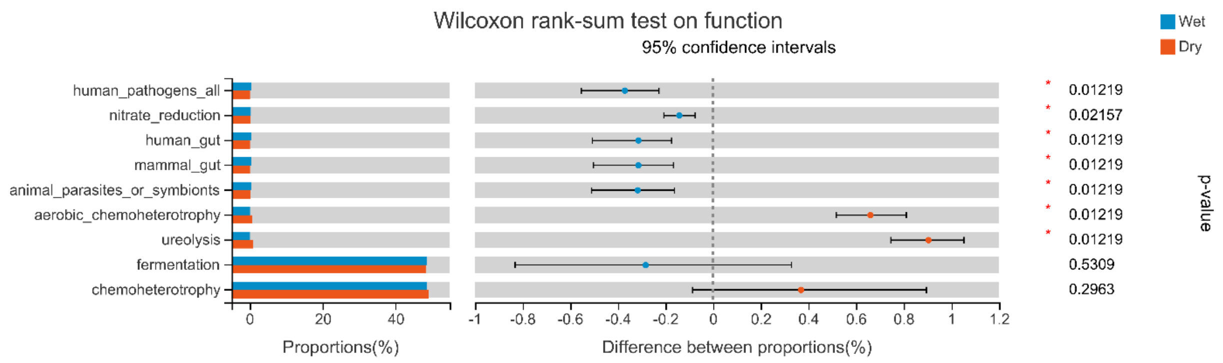

Figure S3. FAPROTAX function prediction. FAPROTAX is an artificially constructed database that maps prokaryotic taxa (e.g., genus or species) to metabolic or other ecologically relevant functions (e.g., nitrification, denitrification). The abscissa represents the function name, the ordinate represents the percentage value of the abundance of a certain function in the sample, and different colors represent different groups. On the far right are P values, $*0.01 < P \le 0.05$.

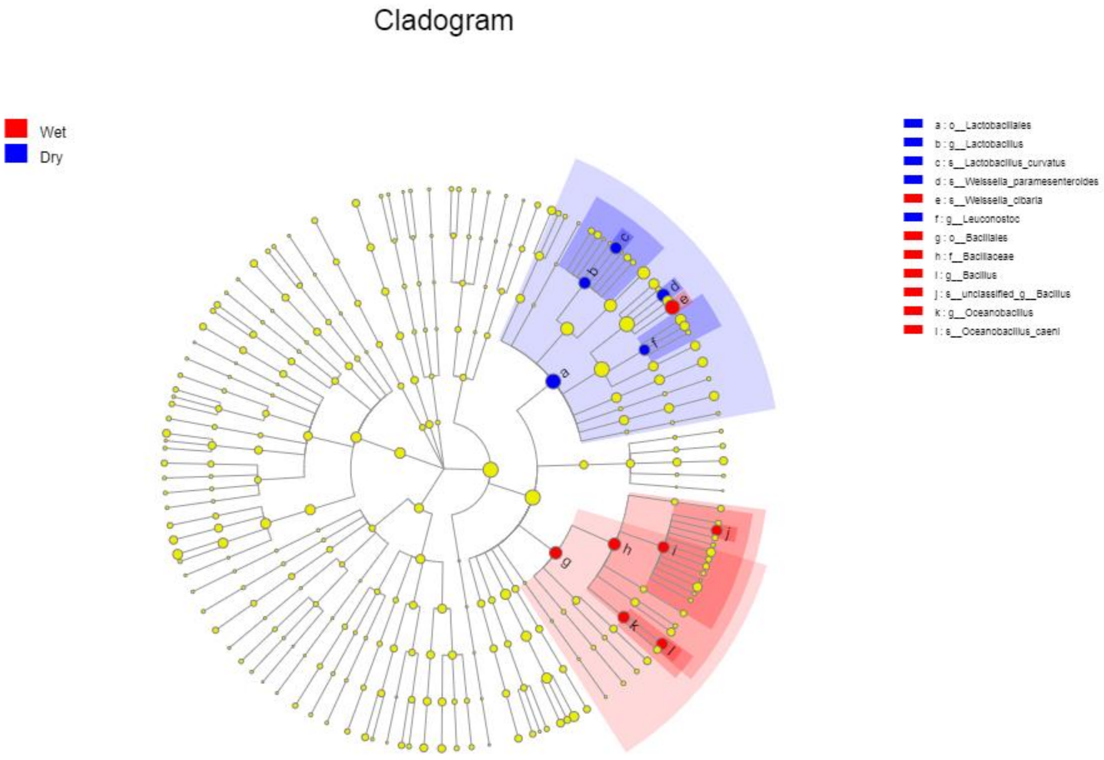

Cladogram Bacteria Part

Figure S4. Comparison and analysis of the relative differential abundance of bacterial microbiome showed in Cladogram. Difference in both the groups (n = 5) are shown as colors. Cladogram through LEfs Show the most abundant taxa in all three mice groups. The size of the circle represents the relative abundance i-e effect size while the different colour represents different group as red indicate wet group, blue colored represents tax in dry samples.

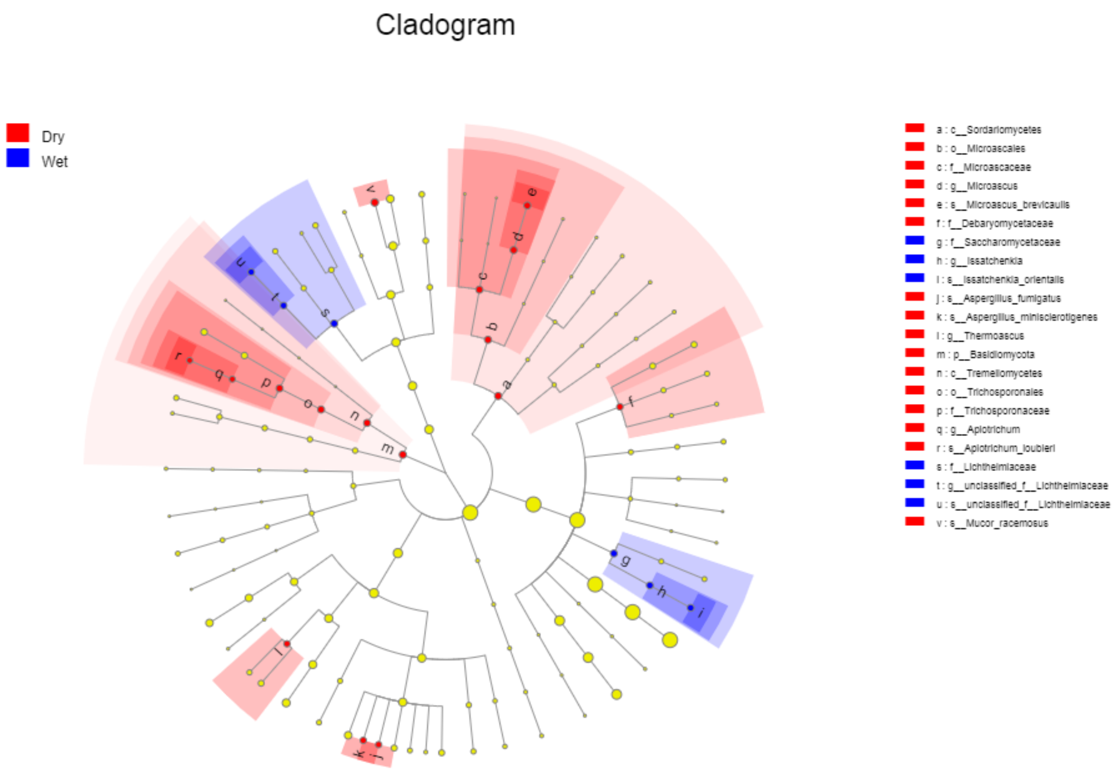

Cladogram Fungi Part

Figure S5. Comparison and analysis of the relative differential abundance of fungal microbiome in Cladogram. Difference in colors represent variation in both the dry and wet groups (n = 5). Cladogram through LEfs show the most abundant taxa in all three mice groups. The size of the circle represents the relative abundance i-e effect size while the different colour represents different group as red indicate wet group, blue colored represents tax in dry samples.

Figure S6. Few Representative pictures of Daqu

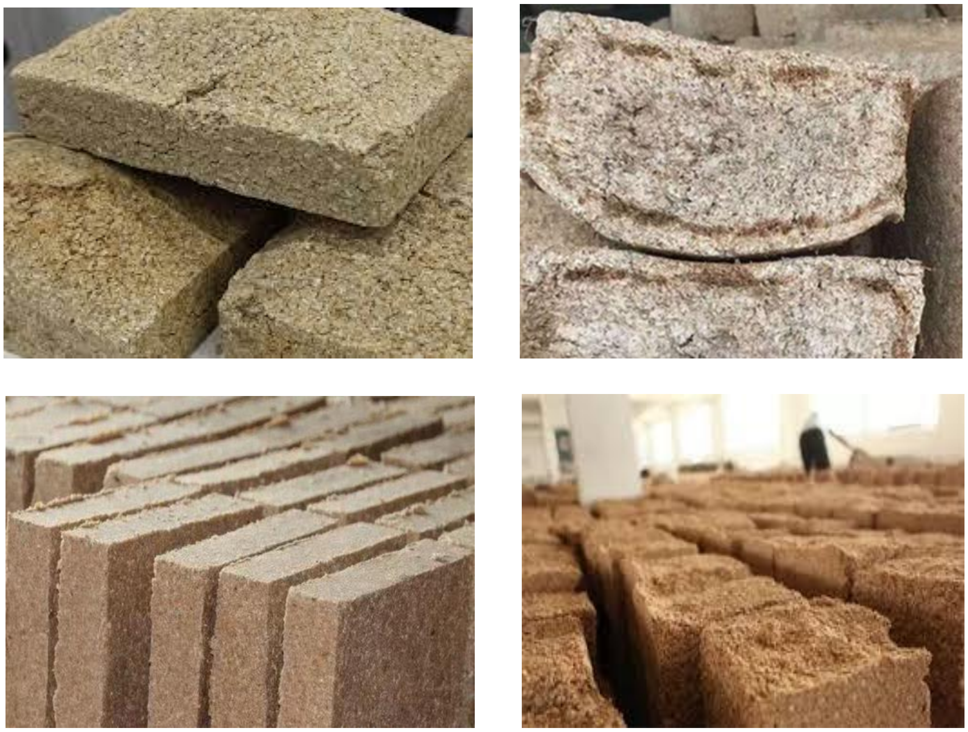

Supplement: Supplementary file 1 [file foods-12-00025-s001.zip › foods-2057755-supplementary.pdf]
